# Structural Heterogeneity of Mitochondria Induced by the Microtubule Cytoskeleton

**DOI:** 10.1038/srep13924

**Published:** 2015-09-10

**Authors:** Valerii M. Sukhorukov, Michael Meyer-Hermann

**Affiliations:** 1 Department of Systems Immunology and Braunschweig Integrated Centre of Systems Biology, Helmholtz Centre for Infection Research, Inhoffenstr. 7, 38124 Braunschweig, Germany; 2 Frankfurt Institute for Advanced Studies, Goethe University of Frankfurt am Main, Ruth-Moufang-Str. 1, 60438 Frankfurt am Main, Germany; 3 Institute for Biochemistry, Biotechnology and Bioinformatics, Technische Universität Braunschweig, Langer Kamp 19b, 38106 Braunschweig, Germany

## Abstract

By events of fusion and fission mitochondria generate a partially interconnected, irregular network of poorly specified architecture. Here, its organization is examined theoretically by taking into account the physical association of mitochondria with microtubules. Parameters of the cytoskeleton mesh are derived from the mechanics of single fibers. The model of the mitochondrial reticulum is formulated in terms of a dynamic spatial graph. The graph dynamics is modulated by the density of microtubules and their crossings. The model reproduces the full spectrum of experimentally found mitochondrial configurations. In centrosome-organized cells, the chondriome is predicted to develop strong structural inhomogeneity between the cell center and the periphery. An integrated analysis of the cytoskeletal and the mitochondrial components reveals that the structure of the reticulum depends on the balance between anterograde and retrograde motility of mitochondria on microtubules, in addition to fission and fusion. We propose that it is the combination of the two processes that defines synergistically the mitochondrial structure, providing the cell with ample capabilities for its regulative adaptation.

Mitochondria are prolonged organelles whose role in controlling key pathways responsible for cellular viability has become well established. Interest in these organelles is constantly revived by ongoing discoveries of their central role in widespread pathologies like diabetes, ischemia, neoplastic, immune and neurodegenerative disorders^1^,^2^,^3^,^4^,^5^,^6^. In all these diseases prominent alterations of mitochondrial morphology are noted^7^.

Under healthy conditions, mitochondrial motility is imperative for cell survival and is one of the main means by which the mitochondrial function is adapted to environmental challenges and is sustained in the long-term^8^,^9^,^10^. The dynamics is believed to proceed through intermittent division (fission), physical motion, eventual reshaping and fusion of particular organelles to the same or unrelated partners. The fission and fusion reactions are executed by specific protein complexes assembled on the surface of mitochondria from local and cytosolic components and involves assistance from the endoplasmic reticulum^11^,^12^. With fluorescent microscopy, the complexes are visible as distinct spots in the mitochondrial membrane, and their finite number exposes a limit on mitochondrial fission capacity. When maximal fragmentation is induced by genetic manipulation, the dimension of the vesicle-like mitochondria is comparable to the original diameter of their tubules^10^,^13^. In the opposite limit, a networked super-fused state of the chondriome may be induced experimentally by restricting fission or enhancing fusion^14^. Besides the availability of the membrane-bound complexes, the fusion reaction is controlled by physical positioning of mitochondria in close apposition to each other.

Cytoskeleton fibers all serve in providing mechanical support for the cell body. Microfilaments and microtubules (MT) are used in addition as tracks for positioning and redistribution of cargos actively transported over the cellular cytoplasm, including mitochondria. Furthermore, the endoplasmic reticulum, mitochondria and other large organelles require the cytoskeleton for the maintenance of their internal architecture^15^,^16^. Complete disruption of the cytoskeleton induces drastic changes in the mitochondrial morphology, associated with a decline of function^17^.

In the majority of animal cell types, the MT component of the cytoskeleton represents a star-shaped array, where each MT is anchored to the centrosome, a proteinatious body available as a single copy during the interphase of the cell cycle^18^. The centrosome serves as an organizing center, at which MTs are nucleated to form tubulin polymers and where the number of MTs is controlled. The opposite MT end often reaches the cell periphery where it can interact physically with the cellular cortex. In the interphase, this interaction promotes positioning and maintenance of the centrosome close to the geometric center of the cell^19^. The morphology and connectivity of the mitochondrial network physically depends on these details of the intracellular organization of the cytoskeleton^15^.

In the longer term, the dynamics of the mitochondrial network redistributes and remixes mitochondrial material within the cellular cytosolic compartment. It leads to the formation of a constantly remodeled partially interconnected network spread over the whole cell and easily observable by optical microscopy^20^,^21^. Because the network impediment causes deterioration of cell function, often followed by death, rigorous decoding of its organizational principles is of urgent priority. While the buildup of mitochondria has been characterized extensively on the small-scale and molecular level, still lacking is an accurate theoretical description of their cell-wide architecture. One of the main obstacles is posed by an immature conceptual basis, not developed enough for a quantitative formulation of the mitochondria geometry, its extraordinary variability of semi-reticular configurations^22^,^23^, as well as its physical dependence on the geometry of the cytoskeleton^15^.

A graph theoretical formulation (Fig. 1 and Methods) was shown to quantitatively capture the mitochondrial morphogenesis^24^. The initial approach, however, ignored inhomogeneity of the intracellular environment and interaction with other organelles, potentially able to strongly modify mitochondrial dynamics or to introduce an additional level of complexity into its network architecture. Here, we explicitly explore the physical dependence of mitochondria on the cellular cytoskeleton^15^. The geometrical characteristics of the MT cytoskeleton are derived from properties of single polymer fibers. The cytoskeleton-dependent multi-scale model is then used to examine the spatial arrangement of mitochondria dictated by their dynamics on the structured background of the MTs. The results provide a deeper understanding of the mechanisms shaping the mitochondrial network in a space resolved manner.

## Results

### Fusion and fission capacity

Mitochondria form an irregular spatial network. Their dynamics consists of physical motion under the action of motor proteins connected to the cytoskeleton (CS)^15^,^17^, accompanied by occasional fission and fusion events. The latter are performed enzymatically by mitochondrial fission/fusion complexes (FFC) able to catalyze the reactions independently of the CS, provided that the fusing organelles are in physical apposition^17^. The overall dynamics known from experiments, thus, involves CS-independent factors along with those imposed by the CS organization^12^.

The model accommodates this distinction by incorporating a CS-independent action of the FFCs, set to operate on top of the CS background. Experimental studies reveal^8^ that FFC components are either distributed uniformly in the membranes of mitochondria or are soluble in the cytosol. In the model, the FFC activity is described by the parameter *γ* defined as the ratio of fusion to fission propensity. It reflects the relative strength of biochemical pathways performing fusion and fission^8^. Quick protein diffusion in the cytosol, enhanced by active mixing of mitochondrial material implies that the enzymatic factor *γ* can be considered as independent of the position inside the cell at steady state.

### Constraints of mitochondrial motility induced by the cytoskeleton

The model assumes that the attachment to the CS determines the position of mitochondria in the cytosol. The actual layout is set by the arrangement of CS fibers as well as by the distribution of the organelles over them. Both factors are able to induce the mitochondria inhomogeneity. It is formulated using density functions depending on the spatial coordinate **r** and the position-independent parameter **ξ**: *Q*_1_(**r**, **ξ**) density of CS-attached mitochondria (measured in units of graph edges), *Q*_2_(**r**, **ξ**) density of mitochondria pairs superimposed at the same position of a CS fiber, *S*_11_(**r**, **ξ**) density of two-fiber crossings occupied by mitochondria on both filaments.

Although the enzymes performing fusion and fission reactions are highly specific for either fission or fusion, the cells are not known to express protein species specialized for either sequential fusion or branching processes^8^. In the model, we put forward the spatially structured CS background as the key discriminator between branching and sequential transformations.

**Figure 1:**
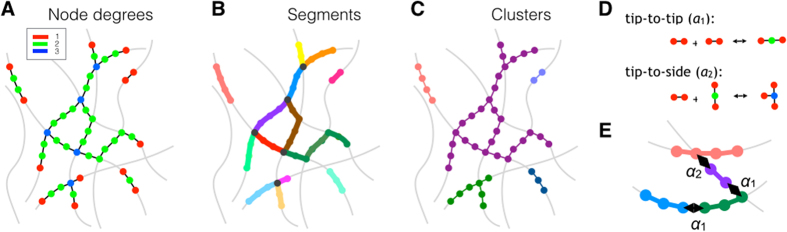
Graph representation of the mitochondrial reticulum and its dynamics. (**A**–**C**) The reticulum can be characterized using different levels of organization: As a collection of nodes of degrees 1 (*red*), 2 (*green*) and 3 (*blue*), i.e. the free ends, bulk nodes and branching nodes, respectively (**A**), linear segments between two nodes of degree 1 or 3 ((**B**) colors distinguish segments), or sets of physically connected segments denoted as clusters ((**C**) colors distinguish clusters). The *grey lines* represent cytoskeleton filaments. (**D**) The nodes are set to undergo two types of fission and fusion transformations^24^: “tip-to-tip” (Eq. (3) with the relative fusion/fission rate constant *α*_1_) and “tip-to-side” (Eq. (4) with the relative rate constant *α*_2_). (**E**) The mitochondria tip-to-tip and tip-to-side reactions are coupled by the availability of cytoskeleton fibers (*grey*) and their crossings. Both, linear MT segments and crossings contribute to the tip-to-tip fusion rate *α*_1_ (the two lower interactions). The branching process *α*_2_ is limited by the availability of the crossings, because it requires at least two sufficiently proximal fibers (upper interaction).

Formation of a mitochondrion branching node necessitates at least two closely positioned (or crossing) MTs. Sequential fusion may occur either between mitochondria located on the same fiber or on different ones, if these happen to be close enough (Fig. 1E).

In a previously published graph theoretical model of mitochondria dynamics without spatial resolution^24^, the constants *α*_1_ and *α*_2_ were introduced to describe the rates of tip-to-tip and tip-to-side fusion events, respectively (Fig. 1 and Methods). With the help of the densities *Q*_2_ and *S*_11_, these rates can be extended to depend on the position in the cell:
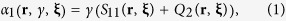

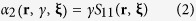
Eqs. (1) and (2) inherently allow coupling the graph theoretical description of mitochondria dynamics to the cellular organization of the CS.

### Mitochondria distribution with respect to the cytoskeleton

Mitochondria are driven in retrograde and anterograde directions along the MTs by dynein and kinesin motor proteins respectively^15^. In the model, their action is described at steady state by a probability distribution *ε* of mitochondria mass *M* = const along the MT contour length *ρ*. The occupancy distribution is derived from a description of the mitochondrial motility along a single MT by a diffusion equation with drift (Eqs. (5) and (6)). The drift is a convenient way to account for eventual difference in the activity between the two motor types^25^. The resulting distribution *ε*(*ρ*, *ψ*) is then governed by ratio *ψ* of the drift velocity to the diffusion coefficient. If the directional bias is absent (*ψ* = 0) the distribution is uniform. When unbalanced (*ψ* ≠ 0), the motor proteins induce an exponential increase or decrease of the mitochondrial density in the direction of the drift (Eqs. (5) and (6)).

### A spherically symmetric cell

**Figure 2:**
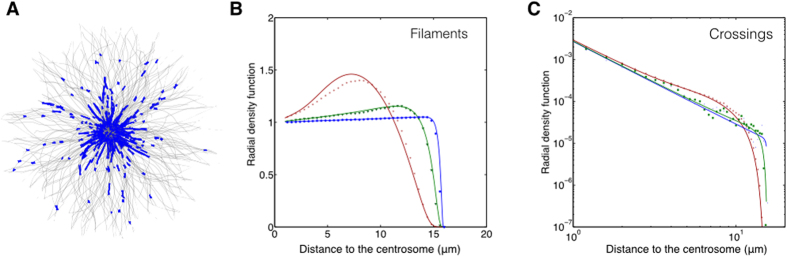
Centrosome-organized microtubule cytoskeleton. (**A**) Configuration of the cytoskeleton in a spherically symmetric cell generated with the Monte Carlo procedure (see Methods). The cell contains 300 MTs (*grey lines*) with a persistence length of 32 μm and a contour length of 16 μm. MT crossing points are shown as *blue dots*. (**B**,**C**) Analytical approximation to the semiflexible polymer model (see Methods) for (**B**) the single-fiber radial density of the MTs (*Q*_0_(*r*)/*n*_f_, Eq. (8)) and (**C**) their crossing density (*S*_00_(*r*)/*n*_f_^2^, Eq. (9)) (*lines*). These are compared to the results of the explicit Monte Carlo representation of the cytoskeleton (*markers*, averaged over 100 randomly seeded runs). The persistence length values are *L*_p_ = 10 μm (*red*), 32 μm (*green*), 100 μm (*blue*). The MT length is *L* = 16 μm and the proximity parameter is *σ* = 0.2 μm.

In this report, an idealized cell configuration with a spherically symmetric MT array is examined (Methods). The arrangement approximates an animal cell, in which the mitochondria-supporting CS consists of the MTs grafted at a centrosome^19^ (Fig. 2A). By utilizing the symmetry, the distance *r* to the cell center is the only spatial coordinate. Upon fixing the position-independent variables other than the motor protein bias *ψ*, the functions *Q*_j_(**r**, **ξ**) = *Q*_j_(*r*, *ψ*), *j* = 1, 2 and *S*_11_(**r**, **ξ**) = *S*_11_(*r*, *ψ*) are simplified and correspond to the radial densities on a sphere shell between *r* and *r* + *dr*. The arrangement is insightful by allowing an analytical derivation of the density functions and a straightforward construction of a Monte Carlo simulation for the CS.

With a spatial Monte Carlo simulation (see Methods), an array of MTs that spreads from the centrosome is generated using a Worm-Like Chain polymer model (Fig. 2A). In all MTs, position and orientation of one end is fixed, while the other end remains free. Orientations of the grafted ends are set random and spherically uniform. The CS-related densities are calculated from the mesh of virtual MTs.

Mechanical characteristics of MT fibers are parametrized with the persistence length *L*_p_, which is a measure for the stiffness of the fibers. The radial distributions of mitochondria *Q*_1_(*r*, *ψ*) and of their same-MT pairs *Q*_2_(*r*, *ψ*) can be calculated analytically from the persistence length *L*_p_ and the mitochondria directionality *ψ* (Eqs. (5, 6, 7, 8)). These approximations (Fig. 2B *lines*) accurately reproduce MT densities, as shown by comparison with data generated using an explicit Monte Carlo representation of the CS (Fig. 2B *markers*).

The three-dimensional organization of reticular organelles must involve branchings^21^,^24^. In the model, the branchings are formed by a tip-to-side fusion reaction (Eq. (4)). It requires two or more CS filaments occupied by mitochondria to come sufficiently close to each other. This “proximity” is formalized using the threshold *σ* = const, set to the mitochondria diameter: whenever the axes of curvilinear cylinders representing the organelles are positioned closer then *σ*, a branching or linear fusion event can potentially occur. The density *S*_11_(*r*, *ψ*) of mitochondria occupying the MT crossings can be calculated from the density *Q*_1_ and a geometric factor accounting for *σ* (Methods, Eq. (9)). Unlike *Q*_1_(*r*, *ψ*), the crossing density *S*_11_(*r*, *ψ*) decreases sharply with the distance to the cell center (Fig. 2C *lines*). This difference of the two functions is a major factor contributing to the network organization of mitochondria.

### Fission and fusion dynamics of mitochondria

As reported previously^24^, fission and fusion processes (Eqs. (3) and (4)) induce steady state configurations of mitochondria reticulum. This remains true for the space-resolved model (see Methods). The dependence of the network fission/fusion rates *α*_1,2_ = *α*_1,2_(*r*, *γ, ψ*) (Eqs. (1) and (2)) and of the mitochondrial density *Q*_1_(*r*, *ψ*) (Methods, Eqs. (5, 6, 7, 8, 9)) on structural constraints by the CS and motor proteins does not change the dynamic laws (Eqs. (10, 11, 12)), but structures the mitochondrial geometry as a function of the distance *r* to the cell center. For any total mitochondrial mass *M*, Eqs. (10, 11, 12) yield a radially resolved geometry of the chondriome, parametrized by the position-independent fission/fusion rate constant *γ* and the occupancy bias *ψ*.

### Chondriome characterization

**Figure 3:**
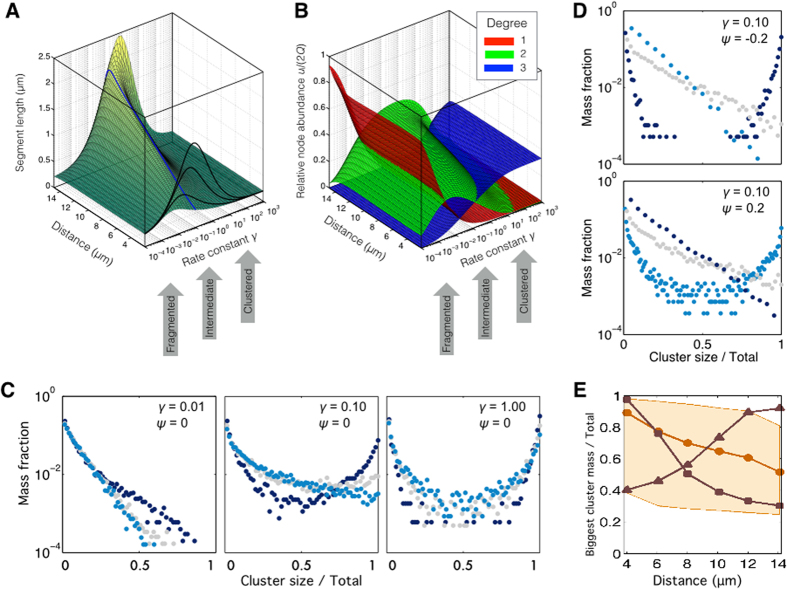
Network structure of the mitochondrial reticulum. (**A**) Length of mitochondria segments obtained as solutions of the graph-based model (Eqs. (10, 11, 12)) in dependence on the radial distance and the fusion to fission ratio *γ*. *Black lines* are cross-sections at distances of 4, 8, and 12 μm from the cell center. The *blue line* is at *γ* = 0.1. (**B**) Node abundances as fractions of the total system size 2*Q*_1_(*r*) with the same dependencies as in A: free ends *u*_1_ (*red*), bulk nodes *u*_2_ (*green*) and branching nodes *u*_3_ (*blue*) are obtained as solutions of Eqs. (10, 11, 12). (**C**) Distribution of the cluster sizes assuming uniform mitochondrial occupancy of the MTs (*ψ* = 0) for *γ* = 0.01 (*left*), 0.1 (*center*) and 1 (*right*) at a distance of 4 (*dark blue*), 8 (*grey*), and 12 μm (*light blue*) from the cell center. (**D**) Distribution of cluster sizes for the intermediate fusion/fission ratio *γ* = 0.1 at distances of 4 (*dark blue*), 8 (*grey*), and 12 μm (*light blue*) from the cell center, assuming a finite drift of mitochondria towards the centrosome (*ψ* = −0.2, *upper panel*) and towards the cell periphery (*ψ* = 0.2, *lower panel*). (**E**) Radially resolved fraction of the largest cluster in the chondriome for a perinuclear (*ψ* = −0.2, *rectangles*), a neutral (*ψ* = 0, *circles*) and a peripheral (*ψ* = 0.2, *triangles*) accumulation of mitochondria along the MTs. An intermediate fusion/fission rate constant *γ* = 0.1 was used (*solid lines* and *marks*). For a balanced radial drift (*ψ* = 0), the sensitivity to the fusion to fission ratio is shown as shaded area by varying between *γ* = 0.01 (*lower edge*) and *γ* = 1 (*upper edge*). Results in (**A**,**B**) are generated with the deterministic model of mitochondria, and in (**C**,**D**) with the stochastic model.

Solutions (Fig. 3A,B) of the deterministic model (Eqs. (10, 11, 12)) characterize the network on the level of nodes *u*_i_(*r*, *γ*, *ψ*) with node degree *i* = 1, 2, 3 (Fig. 1A) and segments (defined in Fig. 1B). A characterization by physically disconnected clusters (defined in Fig. 1C) requires a more detailed stochastic formulation, achieved here with an agent-based simulation of the same system. In the latter, mitochondria are represented explicitly in a virtual cell and subjected to fission and fusion events corresponding to Eqs. (3) and (4). (Methods, Stochastic model of mitochondrial reticulum).

Mitochondrial morphologies observed *in vivo* are cell type-specific and may vary strongly depending on the physiological state^23^,^26^. In the model, the variability can be reproduced by alteration of appropriate parameters as discussed in the following. To highlight the differences, mitochondrial configurations are also compared to those expected in a representative mammalian cell of moderate size and uniform MT occupancy (Reference configuration, see Methods)^21^,^27^.

### Average and radially resolved length of segments

Image analysis of mitochondrial networks suggests that the degree of nodes (Fig. 1A) is restricted to three or less^24^. This implies that the network at position *r* carries (*u*_1_(*r*) + 3*u*_3_(*r*))/2 segments, yielding the mean segment length *s*(*r*, *γ*) = 2*Q*(*r*)/(*u*_1_(*r*, *γ*) + 3*u*_3_(*r*, *γ*)), which can be compared to experiment.

Mitochondria visible on experimental images produced with optical microscopy retain an elongated shape also in perinuclear regions where the reticulum is the densest, suggesting that the segment length does not drop significantly below 1 μm^21^,^28^. In the cell periphery, mitochondria were reported to be a few micrometers long^29^. In the reference parameter set, this radial dependence of *s* is reproduced by the model (Fig. 3A).

### A subtle balance between fission and fusion

There is experimental evidence for an important role of pathways controlling the fission and fusion activity in setting the morphology of mitochondria^13^,^14^. Our results fully support this view: the cell-averaged length of mitochondria *s*(*r*, *γ*) was found highly sensitive to alterations in the global rate constant *γ* which parametrizes the intensity of fusion relative to fission (Fig. 3A). It depends on *γ* in a unimodal way with a peak at *γ*^max^(*r*) (Fig. 3A). Physiological cell-averaged values of a few micrometers^28^ are found in the vicinity of *γ*^max^. The model predicts that mitochondria configurations of healthy cells are only found in a confined range of *γ*. Outside of this range, configurations typical for cells with disturbed fusion and fission balance are obtained (see below). In addition to these clear signatures, the theoretical investigation opens the chance to examine responses of the mitochondrial network to more gentle perturbations and with details not yet possible experimentally.

When the enzymatic propensity for fusion is set small relative to fission (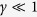, Fig. 3A,B *left arrow*), both the branching and the fission capacities are limited by the absence of bulk nodes (*u*_2_ ≈ 0, Fig. 3B *green*). The in silico node distribution reveals that besides *u*_2_, the network is devoid of the branchings *u*_3_ (Fig. 3B *blue*), but has plenty of free ends *u*_1_ (Fig. 3B *red*). Hence, the chondriome is maximally fragmented, being dominated by short separated organelles everywhere in the cell.

In an idealized system consisting of unbranched segments, an exponential size distribution of disconnected components (clusters) is expected^24^. Indeed, the distribution obtained for 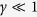 with the agent-based simulation (Fig. 3C *left panel*) is close to exponential and only weakly depends on the distance to the centrosome. This confirms that everywhere in the virtual cell the tiny mitochondria rarely consist of more than one segment. Such extreme mitochondrial fragmentations are often observed in real cells as a reaction to excessive energetic stress or oxidative damage^30^.

With an increased propensity for fusion in the order of *γ*^max^ (*γ* ≈ 0.1, Fig. 3A,B *middle arrow*), the bulk nodes *u*_2_ are created from the free ends by sequential fusion (Eq. (3)) resulting in their dominance (*green* in Fig. 3B). Abundant bulk nodes are equivalent to long mitochondria segments *s* ≈ *s*(*γ*^max^). Longer segments allow for branching via tip-to-side fusion, consuming *u*_2_ (Eq. (4)). However, the branching rate is not limited by the bulk nodes but by the availability of crossings provided by the cytoskeleton (Eq. (2)). In this regime, the segment lengths frequently found in experimental studies on healthy cells are recovered^21^,^27^,^28^,^29^.

For still higher values of *γ*, the segments become smaller again. Long segments are divided by the increasingly abundant branching points produced by tip-to-side fusions (Fig. 3A,B *right arrow*). Additional branchings physically link the mitochondria, which increases the cluster sizes. Despite the small segment lengths, the reticulum is maximally fused and consists of essentially a single supercluster, apart from tiny satellites occasionally popping out. The separation of mitochondria into two distinct size pools (the huge and the tiny) is evidenced by a bimodal shape of their cluster size distribution (Fig. 3C *right panel*). Elevated fusion represents an adaptive response to a mild metabolic stress^31^,^32^,^33^.

### Large-scale partitioning of mitochondria

The model results above suggest that the mechanisms limiting the segment lengths on each side of the peak centered at *γ*^max^(*r*) are different: Branching is active for 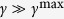 and promotes mitochondrial clustering in contrast to the sequential process prevailing at small *γ*. For intermediate values, the mitochondria structure is sensitive to the MT ordering which controls the branching capacity by setting the density of crossing spots (Eq. (2)). Here, under the influence of the CS, cells may become spatially divided into volumes dominated by distinct types of the reticulum.

**Figure 4:**
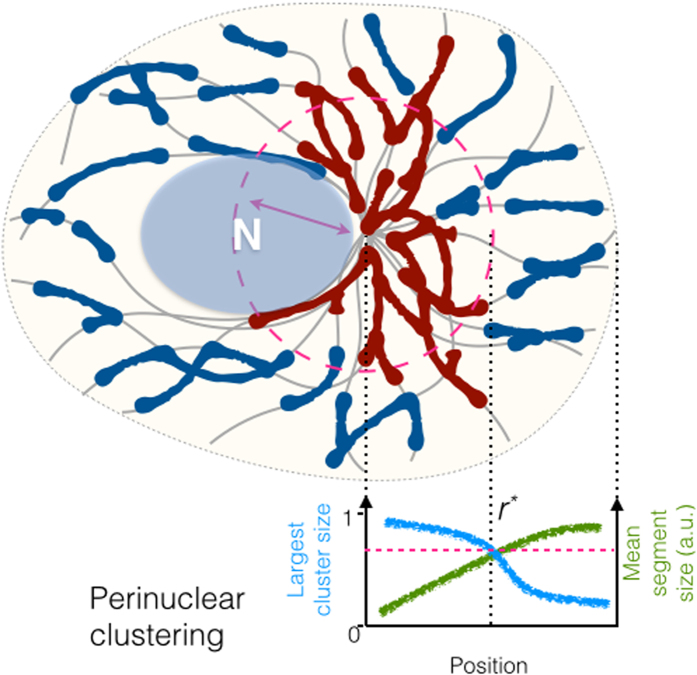
Schematic representation of the mitochondrial reticulum based on the graph model. An uneven density of MTs (*grey*) arranged around the centrosome results in more crossings in the perinuclear region in comparison to the cell periphery and induces a radial gradient of structural parameters of the mitochondrial network. This can lead to the formation of a percolating supercluster (*red*), co-existing together with dispersed mitochondrial fractions (*blue*). The supercluster arises wherever the branching rate of the reticulum dynamics becomes sufficiently large to make the size of the network components exceed a critical distance (*magenta dashed line* and cell region highlighted by the *magenta arrow*). If mitochondria tend to accumulate in the central region or are spread uniformly along the MTs (*ψ* ≈ 0), the supercluster encompasses the perinuclear volume. In the cell periphery mitochondria are much less branched and more fragmented.

Indeed, in configurations with neutral or negative occupancy bias *ψ*, perinuclear mitochondria, characterized by dense MT crossings (Fig. 2A,C), are found branched but highly fused (*dark blue* in Fig. 3C *middle panel* and D *upper panel*), while the peripheral mitochondria, which encounter sparse MT crossings, are mostly linear but rather fragmented (*light blue* in Fig. 3C *middle panel* and D *upper panel*). Accordingly, the chondriome is partitioned into two distinct fractions: A perinuclear supercluster and scattered mitochondria in the periphery, as schematically shown in Fig. 4. The virtual border (Fig. 4 *dashed line*) separating the two regions is characterized by a transition from the dominance of branching nodes to that of bulk nodes. The radial position of the border grows monotonously with *γ*, and can exceed the cell dimension if fusion becomes strong enough.

Thus, in addition to the control of whole-cell averaged characteristics by the balance *γ* of fusion and fission, the spatially resolved formulation reveals a strong sensitivity of the network structure to the radial position inside the cell. A rich heterogeneity of intracellular configurations is shaped by the CS. It would be interesting to identify a way in the model, how to control the intra-mitochondrial variability in space without affecting the chondriome-wide averages. If this was possible, pathways other than the classical fission/fusion mechanism might be essential for a proper arrangement of the mitochondria.

### Fine-tuning the mitochondrial heterogeneity by anterograde and retrograde motility

The intracellular gradient of the CS parameters suggests that the cell-wide network is also reshaped by shifting the mitochondria along the MTs. The reticulum would be sensitive to alteration of the ratio between anterograde- and retrograde-directed motion, parametrized in the model with the radial drift to diffusion ratio *ψ*. A moderate increase of the bias *ψ* from −0.2 to +0.2 modifies the occupancy parameter (Methods Eqs. (5) and (6)) and is sufficient for reverting the mitochondria conformation from perinuclear to peripheral clustering (Fig. 3D,E and Suppl. Fig. 2). The strong sensitivity of the spatial organization relies on the nonlinear amplifying effect of the MT crossing density onto the fusion/fission dynamics, Eqs. (10, 11, 12).

The intracellular variability of mitochondria clusters is more sensitive to the radial drift *ψ* (compare Fig. 3E *rectangles* and *triangles*) than to the fission/fusion ratio *γ*, which causes a rather uniform saturation (Fig. 3E *upper* and *lower* borders of the *shaded area*). Uneven positioning of mitochondria over the MTs, when biased towards the cell center, boosts the sharpness of the spatial partitioning of the reticulum (compare Fig. 3E *rectangles* and *circles*). The super-fused part imposed by the dense CS crossings is additionally stabilized against regulation by *γ* and the size variation of the network components is elevated (compare Fig. 3C *middle panel* and Fig. 3D *upper panel*).

The effect of the radial drift *ψ* on the mitochondrial heterogeneity can be illustrated by comparison of the spatially resolved model to a position-independent network developed previously^24^, in which the distributions are spatially uniform. In the latter, the chondriome acquires either a condensed or a fragmented state, depending on the actual value of the branching rate *α*_2_ = const(*r*)^24^. The two states correspond to distinct thermodynamic phases, separated by a percolation transition which occurs at some critical value *α*_2_^*^. However, because *α*_2_ has the same value everywhere in the cell (is a scalar number), the phases do not coexist and the only way of changing the chondriome conformation is via the fusion to fission ratio *γ*.

The radial dependence of *α*_2_ = *α*_2_(*r*) in the CS-bound system Eq. (2) allows for the coexistence of the condensation states (*α*_2_ < *α*_2_^*^ and *α*_2_ > *α*_2_^*^) in the cell, each occupying distinct parts of the volume (Fig. 4). In the balanced cell configuration (*ψ* = 0), this is evidenced by the qualitative contrast between the cluster size distributions at the perinuclear and the peripheral position (Fig. 3C *middle panel*: *dark blue*, bimodal condensed in the center vs. *light blue*, unimodal fragmented in the periphery). The steeper *α*_2_(*r*) is (i.e. when *ψ* < 0), the more pronounced is the stabilizing effect against modulations of the FFC activity. Also, when the radial shift (*ψ* > 0) counteracts that of the MT crossings, the long-range clustering may become induced (peripherally), but requires a mitochondrial density large enough to overweight the branching sparsity (Suppl. Fig. 3).

## Discussion

Over the past years, mathematical models were established as valuable tools advancing the mitochondria physiology^34^,^35^. However, the models based on biochemical data are limited by ignoring the relation between large-scale structures and functionality, which is critical for this organelle^36^,^37^. Development of more accurate formulations is hindered by the poor characterization of the mechanisms responsible for the chondriome network structure. The difficulty is potentiated by the radical diversity of mitochondria conformations among disparate cell types^23^ and by the ability of the mitochondrial network to abruptly change its structure after long periods of stable operation^30^,^31^,^32^,^33^,^38^.

Experimental manipulation of the balance between the enzymatic fission and fusion complexes (FFCs) has revealed their role as major regulators of the mitochondrial size^8^ and inspired computational examination of mitochondrial dynamics^24^,^39^,^40^. However, the available models assume a reduced dimensionality and are not suitable for examination of the geometric conformation of mitochondrial networks. In particular, the factors regulating internal heterogeneity among different parts of the chondriome or the susceptibility to the activity of FFCs remain poorly understood.

These questions are addressed in the current theoretical work, studying the mitochondrial dynamics systemically under the combined action of FFCs and the cytoskeleton (CS). The chondriome is examined in a spherically symmetric cell representing the mitochondria on centrosome-organized MTs. Within the dual mitochondria-CS system, the local density of mitochondria as well as the density of MT crossings is found to affect the chondriome responsiveness to FFCs. Because in the spherical cell configuration both critical parameters change monotonously along the cell radius, the discussion is conveniently simplified. However, the model may be generalized to less regular cell shapes, provided that the CS density distributions corresponding to Eqs. (8) and (9) are available. This is straightforward, because the motility rates (Eqs. (1) and (2)) assume a generic relation between the CS and fission/fusion dynamics, rather than a particular CS geometry. E.g. in the case of a cell spread on a cover-glass, as often used for optical microscopy, a cylindrical symmetry of MTs could be assumed, eventually adjusted further to account for a variable cell thickness.

The model results provide evidence that the FFCs are not the only factors essential for an accurate setting of the mitochondria fragmentation. Centrosome-organized cells possess the capability to regulate their sensitivity to FFCs by shifting the organelles along the radial direction. This may be one of the major reasons for the often observed perinuclear condensation or spread-out of mitochondria towards the cell periphery. Our data imply, that in such cells, both the balance between the anterograde and retrograde activity of motor proteins as well as between fusion and fission are necessary components for the build-up of the mitochondria network. Therefore, the reticulum is best controlled if the two regulative pathways are connected with common feedback loops, as indeed is observed experimentally^41^,^42^. By such a coupling, the radial motility of mitochondria would enable an inhomogenous and highly flexible distribution of mitochondria sensitive to adaptive signals, as characteristic for animal cells^23^.

Renewal of mitochondria is driven by their compositional heterogeneity sensed via the potential gradient across the inner membrane^43^. Because the CS is predicted to modify the diversity through structural remodeling, it may represent a novel factor affecting the renewal of this organelle. Notably, the impact of other such agents, among which is the diffusion of membrane-bound OxPhos components, the strength of their confinement inside the mitochondria cristae and the production of reactive oxygen species is strongly dependent on the level of chondriome fragmentation^44^,^45^,^46^,^47^,^48^. Quantitative understanding of these processes is imperative for successful treatment of age-related pathologies associated with mitochondria^7^,^49^,^50^.

Intriguing is the involvement of the endoplasmic reticulum in initiation of mitochondrial fission as discovered recently^11^. It brings another spatially networked organelle onto the chondriome scene. Both the mitochondria and the ER are associated with MTs, but perform CS-dependent dynamics of their own. Integrative spatial models could lend a rewarding tool for investigation of such complex systems. Our study contributes to their development by presenting the CS and the mitochondria as a unified system.

In conclusion, the current report examined theoretically for the first time the role of external structural factors in shaping the chondriome. The anisotropic organization of MTs was found to decouple the mitochondrial branching activity from the sequential fusion of parallel organelles. Amplified by nonlinearity of the mitochondrial dynamics, the structure of the MT mesh induces a highly non-uniform geometrical composition on the organelle. The relation can nevertheless be studied in an uncomplicated but accurate formulation of dynamic graphs. Taking into account the close association between morphology and physiological characteristics of mitochondria, a quantitative description of their cell-scale architecture may bring therapeutic merits for metabolic, immunological or neurodegenerative pathologies.

## Methods

### Mitochondrial networks can be described by the graph theory

The generic mathematical framework of graph dynamics was found appropriate for irregular, motile, and spatially extended structures recently^24^. In this initial formulation, a well-mixed homogeneous intracellular environment is assumed. The whole-cell mitochondrial reticulum is represented as an evolving graph consisting of nodes linked by edges (Fig. 1A–D). The topology of the graph is based on the analysis of experimental images of mitochondria^24^, which reveal that nodes with connectivity degrees of 1 ≤ *i* ≤ 3 (Fig. 1A) are sufficient for an adequate representation of the mitochondrial network.

Let *U*_i_ denote the set of such nodes indexed by their type *i*. *U*_1_ corresponds to the free ends of mitochondria, *U*_2_ to places in the bulk where a reaction event may occur, and *U*_3_ to three-way junctions (Fig. 1). Assuming that the network evolution consists of fission and fusion^8^, the reactions correspond to elementary node transformations (Fig. 1D) either of sequential type (“tip-to-tip”, parametrized with fusion to fission rate ratio *α*_1_ = *k*_+_/*k*_*−*_)
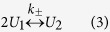
or of branching type (“tip-to-side”, parametrized with the relative rate *α*_2_ = *l*_+_/*l*_*−*_)
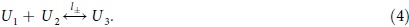


The branching reaction is set here as the simplest possible transformation able to induce the observed 3-dimensional network of mitochondria^24^. The model treats all graph edges equally assigning a constant length to them. The sum of the edge lengths corresponds to the total length of the cellular mitochondria and is an experimental parameter set to a constant. The length of an edge can then be interpreted as smallest mitochondrion unit in the limit of mitochondrial fractionation when the chondriome is dominated by fission.

This previously published graph theoretical approach^24^ is extended here by an explicit representation of the intracellular organization of space with the cytoskeleton.

### Coverage of the cytoskeleton with mitochondria

At steady state, the occupancy 0 ≤ *ε*(*ρ*) ≤ 1 at position *ρ* along the contour of a MT fiber is set by the null condition for the mitochondrial flux at *ρ*: *ψε*(*ρ*) − *dε*(*ρ*)/*dρ* = 0, where *ψ* is the ratio of the drift and diffusion coefficient^25^. If the mitochondrial positioning is restricted to an interval [*l*_1_, *l*_2_], 0 ≤ *l*_1_ ≤ *l*_2_ ≤ *L*, where *L* is the MT length, the integration constant is set by the normalization
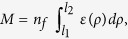
where *n*_f_ = const is the number of MTs and *M* = const is the total length of mitochondria in the cell. Then, the occupancy is distributed either exponentially or uniformly, depending on the presence of the drift,
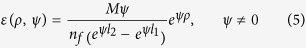

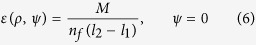
in the interval [*l*_1_, *l*_2_], and is zero elsewhere.

### End-to-end distances of microtubules in a centrosome-organized cell

Predisposition for MT deformation is parametrized with a persistence length *L*_p_, which is the characteristic length of the correlation decay of tangent vectors *∂***r**(*ρ*)/*∂ρ* along the contour *ρ*. On the basis of mechanical characteristics, MTs are classified as semiflexible polymers. Their *L*_p_ is of the same order of magnitude as the full length *L*^51^,^52^.

We consider first the distribution of positions **r** for a point on a MT located at the contour length *ρ* (0 ≤ *ρ* ≤ *L*) away from the grafted end. Due to the spherical symmetry in the initial orientation of the MT ensemble, it is sufficient to consider the distribution *P*(*r, ρ*) of end-to-end distances *r* between the end grafted at the centrosome (taken to be at 0) and the position *ρ* of the point. It can be approximated with the series^53^
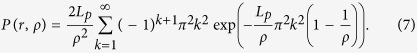
In the calculation of *P*(*r, ρ*), a quick convergence was achieved by splitting the full range of relative distances *r*/*ρ*^53^: The series above was used for *r* ≤ 0.9*ρ*, and its equivalent reformulation in terms of Hermite polynomial for *r* > 0.9*ρ*.

### Radial density of mitochondria

We consider an array of MTs spreading from the centrosome (Fig. 2A). The position of one MT end is fixed at the centrosome while the other end is left free. The orientation of the grafted end is uniformly distributed. Due to the orientational symmetry at the cell center, the radial densities *Q*_j_(*r, ψ*) on a spherical shell *r* of *j* mitochondria attached at the same MT position are proportional to the integral of the single-MT end-to-end distribution *P*(*r*, *ρ*) weighted with the power of occupancy *ε*^j^(*ρ, ψ*) (Eqs. (5–7)):
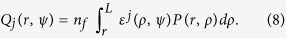


Steric constraints are assumed to limit *j* < 3. It is sufficient to restrict the integration interval to [*r*, *L*] rather than to the full length [0, *L*], because the filament segment *ρ* < *r* does not contribute to *Q*_j_(*r* ≥ *ρ*).

The distribution *Q*_0_(*r*) describes the CS without relation to the associated organelles. It is of general interest as a measure for the spatial configuration used as a backbone for the organelles. Normalized to a single MT, *Q*_0_(*r*)/*n*_f_ is shown in Fig. 2B (*lines*) for a set of MTs with mechanical rigidities parametrized with the persistence length *L*_p_. It adequately approximates the distribution obtained in explicit Monte Carlo Freely Rotating Chain models of MTs (see below) in the regime of semiflexible and stiff structures (*L*/*L*_p_ ≤ 1) representative of MTs *in vivo* (Fig. 2B)^54^.

### Density of mitochondrial crossings

For two-fiber crossings populated with *j* mitochondria on each MT, the density *S*_jj_(*r, ψ*) at a point on the sphere of radius *r* can be found as the mitochondrial mass located inside a sphere of radius *σ* centered at that point, and multiplied by the concentration *Q*_j_(*r, ψ*)
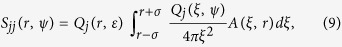
where *A*(*ξ*, *r*) = 2π*ξ*[*ξ* − (*ξ*^2^ − *σ*^2^ + *r*^2^)/(2*r*)] is a geometric factor (see Supplementary Information). Fig. 2C shows the CS-related density function *S*_00_(*r*) (*lines*) along with the data of the Monte Carlo CS model (*markers*). In the bulk of the cytosol we have 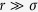, yielding *S*_00_ ≈ *σ*^3^*Q*_0_^2^/(3*r*^2^).

Crossings of three and more MTs involve additional multiplications by the volume density *Q*_j_/(4π*r*^2^) and would fall much steeper than *S*_jj_(*r*). Because their influence would be restricted to the very vicinity of the centrosome, further analysis will be limited to two-fiber crossings.

### Steady state of the mitochondria network in a deterministic formulation

For the spatial extension of the model, the dynamics of the mitochondrial network based on fission and fusion (Eqs. (3) and (4)) is expressed in terms of densities of graph nodes *u*_i_(*r, ψ*), *i* = 1, 2, 3. The steady state of the mitochondria graph is given by the law of mass action^24^
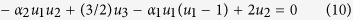

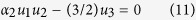
along with the edge conservation condition
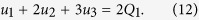


The node densities *u*_i_ are functions of the position *r* and the directionality *ψ* resulting from the dependence of the rate constants *α*_1_ and *α*_2_ (Eqs. (1) and (2)) and the density *Q*_1_ (Eq. (8)) on these parameters. The first two terms of Eqs. (10) and (11) account for the branching reaction (Eq. (4)). The next two terms in Eq. (10) describe the sequential transformation of the mitochondrial graph (Eq. (3)).

### Freely Rotating Chain and Worm-Like Chain polymer models

These models are well established approximations of linear polymers^51^. They are used as representations of single MTs.

In the Freely Rotating Chain (FRC) model, *N* monomer subunits of length *a* each are consecutively connected into a polymer chain to produce the total contour length *L* = *aN*. The FRC scheme assumes that each monomer has a constant bond angle *θ* with respect to the previous one, and a random uniformly distributed torsion angle.

The worm-like chain (WLC) model is a limiting case of the FRC model for *a* → 0, *θ* → 0, *a*/*θ* = const. In this limit, the distance along the polymer contour can be parametrized with a continuous contour length *ρ* (0 ≤ *ρ* ≤ *L*).

### Explicit Monte Carlo representation of the microtubule cytoskeleton

A random space-resolved cellular array of virtual MTs is built explicitly from a set of *n*_f_ linear filaments grafted at the microtubule organizing center with a direction uniformly distributed over a sphere surface. The initial distribution of orientations leads to a quick decrease of the fiber density away from the cell center. The MT radius (12.5 nm) is assumed negligible in comparison to the distance between neighboring fibers^55^. This allows to ignore steric interactions between the MTs and to model them as a one-dimensional differentiable curve **r**(*ρ*) embedded in 3D space and parametrized by the contour length *ρ* (0 ≤ *ρ* ≤ *L*). We explicitly confirmed the latter assumption by comparison with simulations in which a finite MT thickness is applied.

For simplicity, the total length *L* is taken equal for all MTs. Most often, mammalian MTs consist of ≈13 parallel protofilaments forming a hollow tube with a good cylindrical regularity^55^. Taking into account the local axial symmetry, in the explicit simulation, each MT is assembled as a single fiber using the FRC polymer model. The persistence length is related to the bond angle *θ* and the bond length *a* as *L*_p_ = 2*a*/*θ*^2^. The length *a* is set constant and equal to 20 nm. By varying *θ*, the desired persistence length is then obtained.

The model is formulated in the C++ programming language. Pseudorandom numbers are generated using the Mersenne Twister algorithm^56^, as encoded in the boost::random library ver. 1.55 (http://www.boost.org)^57^.

### A stochastic model of the mitochondrial reticulum

The mutual interaction of the nodes in a set of graph edges is calculated with the exact stochastic algorithm of Gillespie^58^. The nodes of degrees 1 ≤ *i* ≤ 3 participate in fusion and fission reactions defined by Eqs. (3) and (4) with parameters by Eqs. (1) and (2). The system is an explicit agent-based representation^24^ of the approximation given by Eqs. (10, 11, 12). By setting the reaction parameters Eqs. (1) and (2) dependent on the cytoskeleton Eqs. (5, 6, 7, 8, 9), network configurations at specific positions along the cell radius, values of global rate constant *γ* or drift parameter *ψ* are generated. Each dataset is an average of 1000 randomly seeded runs and is recorded after full equilibration. Within a run, nodes participating in a particular event (Eqs. (3) and (4)) are chosen randomly with equal probability among the nodes of the appropriate type. Random numbers are generated using VSL routines, part of Intel Corp. (Santa Clara, CA) Math Kernel Library.

### Reference cell configuration

The mitochondria are positioned on 200 MTs (*L*_p_ = 32 μm)^54^ in a spherically symmetric centrosome-organized cell with radius equal to the MT contour length *L* = 16 μm and spatial discretization Δ*r* = 0.01*L*. The crossing threshold *σ* and the edge length of the mitochondrial graph are both equal to 0.2 μm and correspond to a typical diameter of mitochondria. The cumulative reticulum length *M* = 1 mm reflects the one found in insulinoma cells or standardized fibroblasts^13^,^21^,^27^. Mitochondria are distributed uniformly (*ψ* = 0) between the MT contour positions *l*_1_ ≈ 0.5 μm and *l*_2_ ≈ 15 μm (Eqs. (5) and (6)). Unless indicated explicitly, this configuration is applied as the default parameter set.

### General remarks

The analysis and visualization of the output data, as well as the numerical integration and solution of algebraic equations are performed using Matlab (The MathWorks, Inc.).

## Additional Information

**How to cite this article**: Sukhorukov, V. M. and Meyer-Hermann, M. Structural Heterogeneity of Mitochondria Induced by the Microtubule Cytoskeleton. *Sci. Rep.* **5**, 13924; doi: 10.1038/srep13924 (2015).

## Supplementary Material

Supplementary Information
