# Supplementary material for: Structural Heterogeneity of Mitochondria Induced by the Microtubule Cytoskeleton
Source: Sci Rep. 2015 Sep 10;5:13924. doi: 10.1038/srep13924 (PMC4565121; doi:10.1038/srep13924)
Supplement: Supplementary Information [file srep13924-s1.pdf]

Structural Heterogeneity of Mitochondria Induced by the Microtubule Cytoskeleton

Valerii M. Sukhorukov and Michael Meyer-Hermann

Supplementary Information

Density of the microtubule crossings

Because the microtubules are considered independent of each other, we seek the radial density $S(r)$ of the crossing points (Eq. (9) of the main text) over the shell between r and $r+dr$ on a sphere (O, r) (Suppl. Fig. 1). It may be calculated as the probability $Q(r)$ of finding a point belonging to an MT curve anywhere in the shell, multiplied by the probability of finding a crossing partner inside a sphere (Θ, σ) of radius σ centered on the surface at r. The latter probability is equal to

$$\int_{V(\sigma)} \frac{Q(r)}{4\pi r^2} dV = \int_{r-\sigma}^{r+\sigma} \frac{Q(\xi)}{4\pi\xi^2} A(\xi, \sigma, r) d\xi$$

where the integral is taken over the volume $V(\sigma)$ of the second sphere. Because the density field $Q/(4\pi r)$ has the only gradient in the radial direction, one-dimensional integration weighted with the cross-section area A (Suppl. Fig. 1, *black dotted line*) can be applied. A is an area of a cap on the sphere (O, ξ) limited by the plane (Suppl. Fig. 1, *black straight line*) defined by its cross-section with (Θ, σ): $A = 2\pi\xi(\xi - r_0)$. Position $r_0 = (\xi^2 - \sigma^2 + r^2)/(2r)$ of the plane can be found by solving equations for the two spheres.

From this, Eq. (9) of the main text follows.

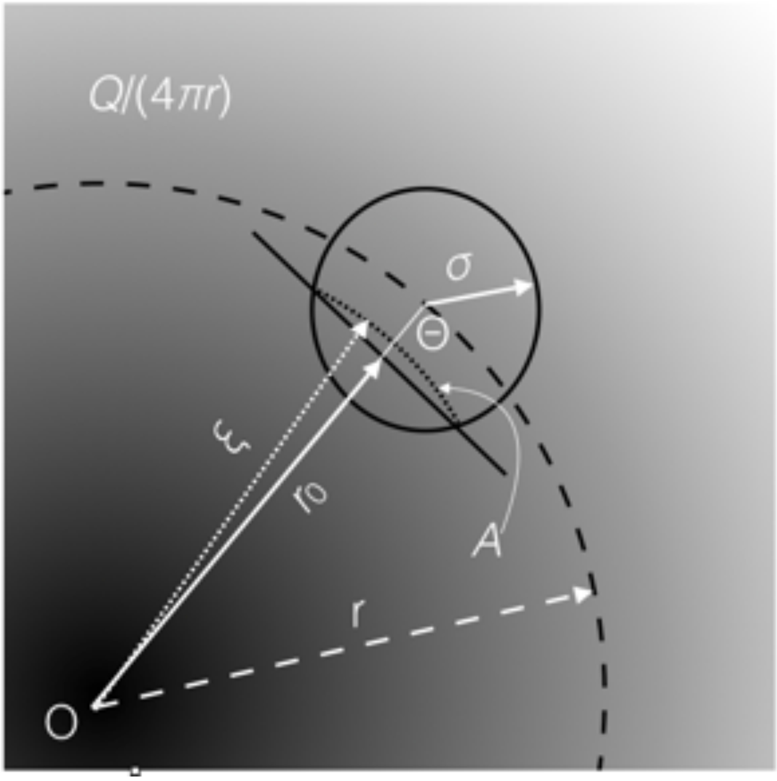

Supplementary Figure 1
Scheme of the geometric configuration for the calculation of the microtubule crossing density.

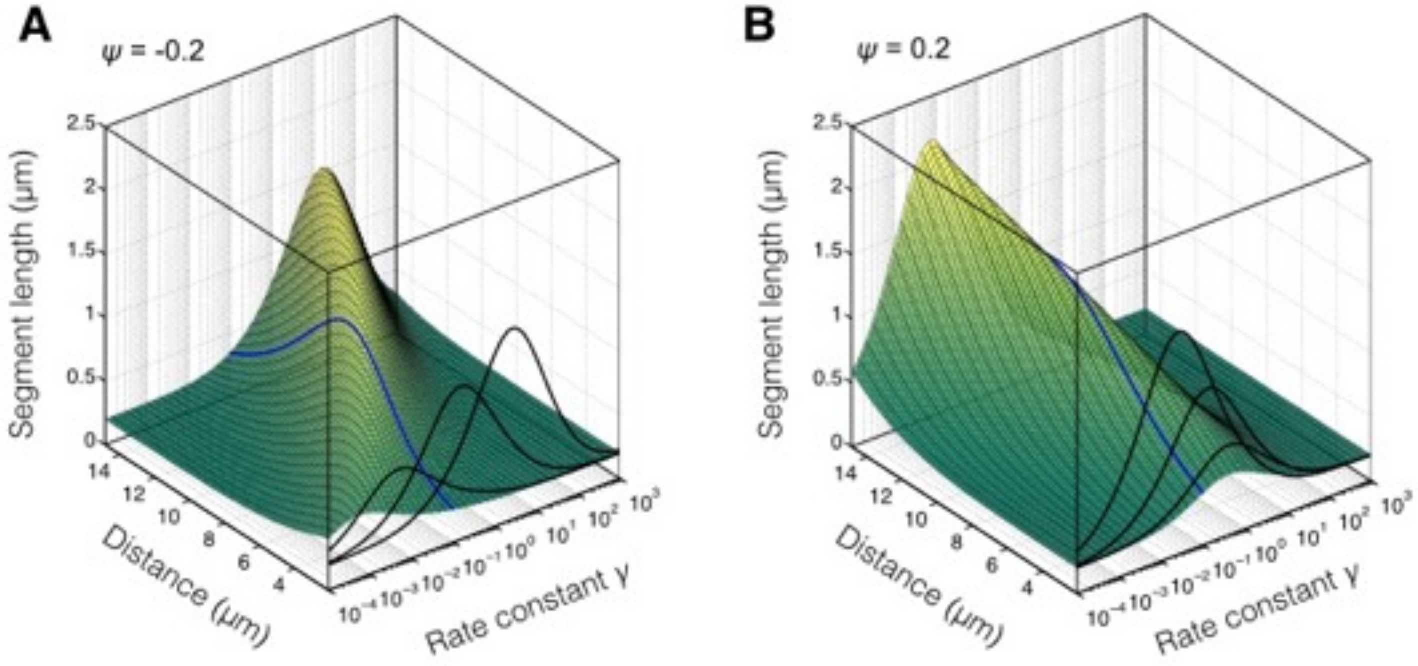

Supplementary Figure 2

Steady-state length *s* of mitochondrial segments obtained within the graph-based model using the reference parameter set (defined in the Methods). A biased mitochondrial transport towards the cell center (A) or periphery (B) is assumed. *Black lines* are cross-sections at distance 4 μm, 8 μm, and 12 μm from the cell center. The *blue line* is at $\gamma = 0.1$. A comparison with the non-biased reference configuration (Fig. 3A of the main text) indicates that the intracellular distribution of the mitochondria geometric parameters is strongly affected by the above modifications, in contrast to their cell-averaged values (close to 1 μm) .

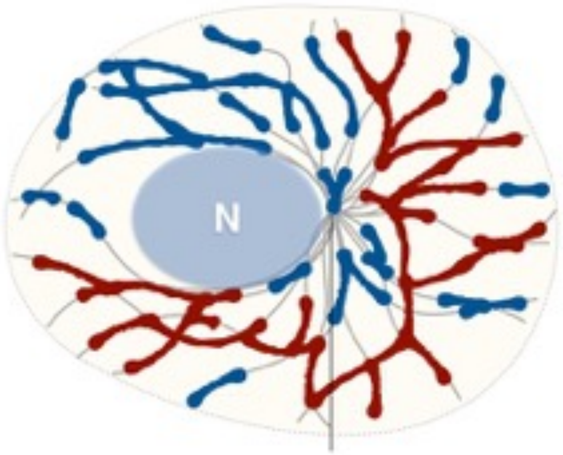

Supplementary Figure 3

If the balance between the retrograde and anterograde motion shifts sufficient mitochondria towards the periphery ($\psi > 0$), the gradient of the reticulum structural parameters opposes the increase of MT occupancy in the outer direction. Volume region with the highest mitochondria connectedness repositions outwards, where the MT crossings are spacer. The reduced branching activity is counteracted by the more intensive sequential fusion. The mitochondria segments are longer than for $\psi = 0$, but their percolation is less favored.
